# Investigation of patient and observer agreement on description of seizures at initial clinical visit

**DOI:** 10.1002/acn3.50950

**Published:** 2019-12-05

**Authors:** Maha N. Saleem, Christopher A. Arencibia, Kevin McKenna, Sabrina Cristofaro, Kamil Detyniecki, Daniel Friedman, Jacqueline French, Hal Blumenfeld, the HEP Investigators

**Affiliations:** ^1^ Department of Neurology Yale University School of Medicine 333 Cedar Street New Haven Connecticut 06520; ^2^ Department of Neurology University of California San Francisco California 94143; ^3^ NYU School of Medicine NYU Langone Comprehensive Epilepsy Center New York New York 10016; ^4^ Department of Neuroscience Yale University School of Medicine 333 Cedar Street New Haven Connecticut 06520; ^5^ Department of Neurosurgery Yale University School of Medicine 333 Cedar Street New Haven Connecticut 06520

## Abstract

There have been few studies of agreement between seizure descriptions obtained from patients and observers. We investigated 220 patients and observers who completed structured questionnaires about patients’ semiological seizure features at the initial clinical visit. Inter‐rater reliability was assessed using Cohen’s kappa and indices of positive and negative agreement. Patients and observers had excellent agreement on the presence of memory impairment and generalized shaking and stiffness during seizures. In addition, patients under‐reported seizure descriptions more easily observed externally, whereas observers under‐reported change in patient location at seizure end. These findings may guide interpretation of clinical histories obtain in epilepsy care.

## Introduction

Patients’ first‐person descriptions of their seizures are often used clinically in diagnosis and treatment of epilepsy. However, self‐reported seizure characteristics may not agree with those described by an external observer. Accurate seizure descriptions are essential for classification of epileptic seizures and clinical decision‐making, and can also give indications about the severity and localization of seizures.^1^, ^2^, ^3^, ^4^ Impairment of conscious awareness, responsiveness, and behavior during seizures can further adversely affect patients’ quality of life including driving safety, employment, social ties, and school performance.^5^ Impaired cognition during and following seizures may also interfere with reliable reporting. An important, unknown piece of information is the inter‐rater reliability between patients and observers on the description of seizures, which can provide important insights into reports typically obtained in a clinical setting.

Previous studies have only investigated patients’ inability to provide accurate report of their seizure frequency and whether they are aware of their seizures^6^, ^7^ or have separately analyzed reports by external observers. Most studies have assessed the accuracy seizure descriptions by witnesses only, and not the inter‐observer rate of agreement between patients and witnesses.^8^, ^9^, ^10^ Benbir et al. investigated the inter‐observer variability between two neurologists and a caregiver, finding overall good concordance which varied for different seizure semiology and characteristics.^11^ Here we examine the inter‐rater reliability of seizure descriptions based on reports from both patients and observers obtained during the initial clinical visit for epilepsy care, a scenario which occurs commonly in a clinical context.

## Methods

### Subjects and data collection

A total of 457 subjects newly diagnosed with focal epilepsy aged 11–75 were enrolled in a multicenter study as part of the Human Epilepsy Project (HEP, http://www.humanepilepsyproject.org/). The study was approved by the applicable institutional review boards, and written informed consent was obtained from all participants. Coordinators administered DISCOVER (Diagnostic Interview for Seizure Classification Outside of Video‐EEG Recording) questionnaires to patients (enrollment requirements were IQ> 70 and ability to fill in questionnaires) and external observers (if available, self‐selected by patients based on being in a position to report) for each patient‐reported seizure type during initial outpatient clinic visits at participating academic medical centers. Observers were in most cases family members or other close contacts available at the time of the initial clinic visit. To confirm the diagnosis of epilepsy and to exclude nonepileptic spells, every subject was reviewed by an independent reviewer at each center who had access to the patient’s relevant clinical data including EEG, video‐EEG (if available), medical records, MRI, and all clinical semiology data obtained from both patients and observers. Nonepileptic spells were identified as best possible, but of course could not be fully excluded when video‐EEG was not done, and the initial diagnosis was thus determined based on all available data as is often the case in clinical practice. If EEG and MRI were abnormal, data were reviewed by one person. If the subject had a normal EEG and MRI, their data were submitted to an independent adjudication committee of 5 experts, and subjects were rejected if the seizure description did not suggest a> 80% certainty (agreement of 4 of 5 committee members) that the events were epileptic seizures.

We analyzed only items from the questionnaires with binary descriptions of seizure‐related deficits (presence or absence of an abnormality), and where comparable items existed on both patient and observer forms (see Data S1). Questionnaires were administered by study coordinators at each site trained to enter an affirmative response for any descriptors endorsed by patients or observers for each seizure type. Note that a response (even if it was “None of the above” or “Unknown/not sure”) was required for each item which enabled us to distinguish negative responses (absence of an abnormality) from cases where an item was simply not completed. For each seizure type, only questionnaire items that were completed in both patient and observer reports were included. Most often incomplete questionnaire items on the observer forms occurred when patients came to the clinical visit alone without an observer, so that of the 457 subjects initially enrolled only 220 subjects had corresponding questionnaire items completed both by patients and observers. Therefore, data from a total of 220 patients were used in the analysis, which included 335 seizure type descriptions (mean of 1.5 seizure type descriptions per patient).

### Statistical analysis

Inter‐rater reliability between patients and observers in the description of seizures was assessed with the Cohen kappa (κ) test. Concordance was rated as “poor” for κ values ≤ 0.2; “fair” for 0.21–0.40; “moderate” for 0.41–0.60; “good” for 0.61–0.80; and “excellent” if κ exceeded 0.81. Because use of kappa alone can be misleading when the relative proportions of responses in “Yes” and “No” categories for the two raters are highly unequal, we combined kappa with two separate indexes of the raters’ positive and negative decisions (see Data S2). The proportion of positive agreement (p_pos_) measures agreement in which both patients and observers respond “Yes,” and proportion of negative agreement (p_neg_) measures agreement where both respond “No.” We then considered inter‐rater reliability to be excellent only if κ, p_pos_, and p_neg_ were all >0.8. A binomial test was used to examine the proportions/percentages of responses that disagreed. Two‐way analysis of variance (ANOVA) with Tukey test of additivity for interaction was used in conjunction with a post hoc Tukey–Krammer test to determine whether patient and observer demographic or clinical characteristics were related to inter‐rater reliability. Correlation analyses including Spearman and Pearson coefficients and r‐square values were used on ordinal or continuous variables as appropriate. A value of *P* < 0.05 was considered statistically significant.

## Results

The 220 patients had an age range of 11–64 (mean 30.4 ± 14.7) years, and 146 patients (66%) were female. The total number of seizures witnessed by the observer for each patient ranged from 1 to >10 (mode 2 to 5 seizures) at the time of the initial clinical visit. Concordance between patients and observers was excellent (κ, p_pos_ and p_pos_ all >0.8) for questions about whether or not patients remember what happens during the seizure (κ = 0.918), and stiffen and shake all over (κ = 0.849) (Table 1). The remaining items had fair to good agreement between patients and observers; the next highest κ values were turning blue with stiffening and shaking (κ = 0.725), and bloody drooling with stiffening and shaking (κ = 0.639). The seizure description from the postictal period (irregular/abnormal/deep/shallow breathing or snoring) showed the least agreement (κ = 0.254).

**Table 1:** Patient and observer responses to descriptive seizure questions.

	Agreement		Disagreement		κ^c^	P_pos_ d	P_neg_ d	Disagreement *P*‐Value^e^
	Patient Yes^a^ Observer Yes^a^ * n* (%)	Patient No^b^ Observer No^b^ * n* (%)	Patient Yes^a^ Observer No^b^ * n* (%)	Patient No^b^ Observer Yes^a^ * n* (%)				
Does not remember what happens during seizure?	189 (73%)	61 (24%)	2 (1%)	6 (2%)	0.918	0.979	0.938	0.289
Stiffens and shakes all over?	126 (41%)	158 (51%)	11 (4%)	12 (4%)	0.849	0.916	0.932	1
Turns blue, stiffens and shakes all over?	22 (18%)	88 (72%)	1 (1%)	11 (9%)	0.725	0.786	0.936	0.006
Bloody drooling, stiffens and shakes all over?	21 (17%)	85 (70%)	5 (4%)	11 (9%)	0.639	0.724	0.914	0.210
Grunting, stiffens and shakes all over?	25 (20%)	74 (61%)	2 (2%)	21 (17%)	0.563	0.685	0.865	< 0.001
Drools during seizure?	75 (22%)	195 (58%)	12 (4%)	53 (16%)	0.562	0.698	0.857	< 0.001
Finds self in a different position/location after seizure?	97 (29%)	161 (49%)	47 (14%)	24 (7%)	0.554	0.732	0.819	0.009
Eyes closed throughout seizure?	31 (9%)	266 (79%)	22 (7%)	16 (5%)	0.554	0.620	0.933	0.418
Falls to ground/loses posture during seizure?	64 (19%)	210 (63%)	37 (11%)	24 (7%)	0.551	0.677	0.873	0.124
Eyes rolled up, stiffens and shakes all over?	46 (38%)	47 (39%)	9 (7%)	20 (16%)	0.528	0.760	0.764	0.061
Mouth movement during seizure?	42 (13%)	237 (71%)	18 (5%)	38 (11%)	0.497	0.600	0.894	0.010
Both sides of the body stiff during seizure?	68 (20%)	192 (57%)	22 (7%)	53 (16%)	0.486	0.645	0.837	<0.001
Drooling, stiffens and shakes all over?	52 (43%)	38 (31%)	5 (4%)	27 (22%)	0.485	0.765	0.704	<0.001
Decreased ability to respond during seizure?	181 (61%)	49 (17%)	21 (7%)	45 (15%)	0.448	0.846	0.598	0.004
Speaks repetitive phrases during seizure?	13 (4%)	294 (88%)	13 (4%)	15 (4%)	0.436	0.481	0.955	0.851
Prolonged groan or scream, stiffens and shakes all over?	14 (11%)	85 (70%)	7 (6%)	16 (13%)	0.435	0.549	0.881	0.093
One hand/arm stiff or in an abnormal posture during seizure?	24 (7%)	261 (78%)	27 (8%)	23 (7%)	0.403	0.490	0.913	0.636
Talks nonsense during seizure?	19 (6%)	275 (82%)	18 (5%)	23 (7%)	0.412	0.481	0.931	0.533
Glassy stare during seizure?	111 (33%)	122 (36%)	29 (9%)	73 (22%)	0.401	0.685	0.705	<0.001
Eyes closed, stiffens and shakes all over?	17 (14%)	77 (63%)	14 (11%)	14 (11%)	0.395	0.548	0.846	1
Back arching, stiffens and shakes all over?	8 (7%)	93 (76%)	1 (1%)	20 (16%)	0.361	0.432	0.899	< 0.001
Purposeless/aimless hand movements during seizure?	37 (11%)	219 (65%)	30 (9%)	49 (15%)	0.334	0.484	0.847	0.042
One side of the body stiff during seizure?	4 (1%)	312 (93%)	11 (3%)	8 (3%)	0.267	0.296	0.970	1
Irregular/abnormal breathing or snoring after seizure?	12 (5%)	186 (77%)	11 (5%)	34 (14%)	0.254	0.348	0.892	< 0.001

a “Yes” or “Always” responses to questionnaire (see Data S1) are combined in Table 1 as “Yes.”

b “No” or “Never” responses to questionnaire (see Data S1) are combined in Table 1 as “No.”

c Inter‐rater reliability represented by κ values are presented in descending order in the Table.

d The Cohen’s κ, proportion of positive agreement (P_pos_) and proportion of negative agreement (P_neg_) were calculated as in Data S2.

e *P*‐values are from binomial test, comparing proportion of patients and observers who say “Yes” or “No” in the Disagreement columns to chance (equal “Yes” and “No” responses).

**Figure 1:**
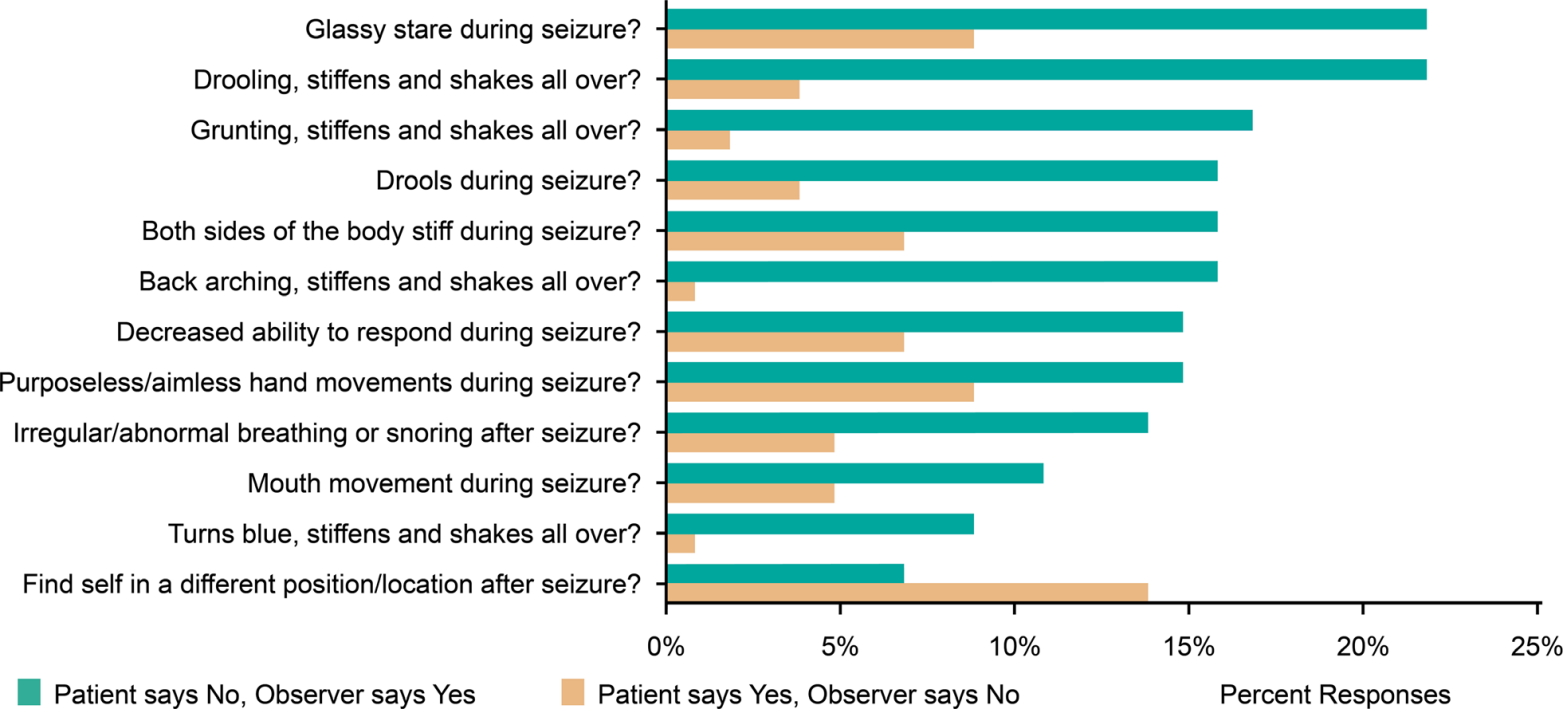
Observers report more abnormal behaviors during seizures than patients. Seizure features where percentage of “Yes” and “No” reports differed significantly between observers and patients (*P* < 0.05, binomial test, Table 1) are shown in descending order of percent “Yes” responses by observers. Observers reported “Yes” more often than patients for 11 of 12 of these seizure features.

Of the 24 questions analyzed, 12 revealed significant differences in the way patients and observers disagreed (Table 1, Disagreement columns and Disagreement P‐values). For most questions with significant differences, observers more often responded “Yes” than patients (Fig. 1). There was only one question in which patients significantly responded “Yes” more often than observers, and this was finding themselves in a different position or location after the seizure (Fig. 1).

Correlation and ANOVA analysis were used to determine whether patient‐related demographics and clinical features or observer‐related parameters correlated with inter‐rater reliability. Of the patient‐related variables, total number of seizures in a lifetime, age, gender, and patient’s reported ability to remember what happens during the seizure did not show significant relationships with κ‐values. Likewise, of the observer‐related data, number of seizures witnessed, and observer’s report on patient’s ability to remember what happens during seizures also did not show significant relationships with increased agreement.

## Discussion

Our study was designed to explore the extent to which patients and observers agree in their descriptions of seizures during the initial clinical visit for epilepsy care. We found excellent inter‐rater reliability between patients and observers in two questions: Does the patient remember what happens during the seizure, and do they stiffen/shake all over. All other behavioral descriptions of seizures showed fair to good level of agreement (Table 1). We also found that when patients and observers disagreed on seizure‐induced impairments, observers reported impairments more commonly than the patients. The descriptions more often reported by observers include both ictal and postictal features. However, finding oneself in a different position/location after the seizure – likely more subjectively and directly experienced by patients than observers – was the only description over‐reported by patients.

The high level of agreement between patients and observers in the two questions related to ictal memory impairment and stiffening/shaking all over suggests these two questions may be very useful for healthcare practitioners to obtain reliable information about seizure severity and to guide clinical care. We also found that patients reported less deficits during seizures than observers (Fig. 1), which could lead to underestimation of seizure severity. This reinforces the importance obtaining a history not only from the patient, but also an observer if one is available.

Additional factors such as patient’s age, gender, and total number of seizures, as well as the observer’s familiarity with the seizures, showed no significant correlation with κ values. In the study of Heo et al., there was no correlation between demographic parameters and the accuracy of seizure descriptions other than the education level of the informant.^9^ Another study demonstrated that age, gender, education level, and the patient–observer relationship did not correlate with seizure notification.^12^

The study has some limitations which should be addressed in future investigations. For example, the DISCOVER form does not include information about seizure duration, frequency, stereotypy, and commonly associated symptoms such as incontinence which could provide useful insights. In addition less than half of patients had forms completed by observers, mainly due to patients arriving at clinic alone; however, it is possible that observers were present in some cases but did not complete forms, a potential source of bias that should be addressed in future work. A nonepileptic control group would be of interest to determine whether the differences in description of events between patients and observers are specific to epilepsy or might also be seen in other paroxysmal disorders such as sleep disorders or movement disorders. The initial clinical visit is also a special setting where important information may not yet be available to patients, observers, or to clinicians. The present cross‐sectional study at initial diagnosis should be supplemented by a longitudinal follow‐up study, which, although limited to the subset of patients with follow‐up, would have several advantages in providing additional important objective data. For example, comparison of semiological reports from patients and observers to objective data from follow‐up video/EEG monitoring could be useful in future studies and would also help confirm seizure diagnosis (epileptic vs nonepileptic), classification, and localization.

The fundamental mechanisms of unreliable reporting are not known and should be studied further. Possible effects of impaired consciousness or memory on seizure descriptions should be investigated, as it has been shown that the inter‐observer agreement differs based on seizure classification.^13^ Impaired consciousness and memory of having seizures (i.e. inaccurate report of whether or not a seizure occurred^7^, ^14^) should be carefully distinguished from impaired consciousness or memory during and after seizures. To further complicate matters, both types of impairment are not necessarily identical with ability to accurately describe whether or not memory impairment was present during a seizure. Although impaired peri‐ictal consciousness and memory may play an important role in affecting patients’ ability to accurately report both the occurrence of and nature of their seizures at a later time, these relationships require further rigorous investigation.^7^, ^14^, ^15^, ^16^, ^17^ In addition, whether patient report of seizure semiology is based on their own recall, or on descriptions they have been told by others is another issue not addressed in the present study. Finally, although at the initial visit the patient and observer reports may be the only information at hand, ultimately neither source may be accurate, so comparison to objective data such as video‐EEG monitoring will be highly valuable in cases where such information is available.

In summary, we found that questions about patients’ ability to remember what happens during seizures, and presence of grand mal shaking had excellent concordance between patients and observers; and that observers reported more overall seizure‐related deficits than patients. Consideration of observations with highest concordance rates may assist in diagnosis and treatment of epilepsy, whereas those with poor agreement should be further investigated and better delineated. Future research should further assess the effect of self‐ and observer‐reporting of patients’ seizures on diagnosis and clinical outcomes.

## Conflict of Interest

Maha Neha Saleem, Christopher Andrew Arencibia, Kevin McKenna, Sabrina Cristofaro, and Hal Blumenfeld report no disclosures. Dr. Kamil Detyniecki has received research support to Yale University for investigator‐initiated studies from Eisai, Sunovion, Acorda, and Upsher‐Smith. Dr. Daniel Friedman receives salary support for consulting and clinical trial related activities performed on behalf of the Epilepsy Study Consortium, a nonprofit organization. Dr. Friedman receives no personal income for these activities. NYU receives a fixed amount from the Epilepsy Study Consortium toward Dr. Friedman’s salary. Within the past year, the Epilepsy Study Consortium received payments for research services performed by Dr. Friedman from: Adamas, Biogen, CuroNZ, Engage Pharmaceuticals, Pfizer, Takeda, and Zynerba. He has also served as a paid consultant for Penumbra. He has received honoraria from Neuropace, Inc for educational materials. He receives research support from UCB, Inc and Empatica. Dr. Jacqueline French receives NYU salary support from the Epilepsy Foundation and for consulting work on behalf of the Epilepsy Study Consortium for Eisai, Lundbeck, Pfizer, Sunovion, and UCB Inc., who also support HEP. Dr. French has also received research grants from Eisai, Lundbeck, Pfizer, Sunovion, and UCB Inc.

## Supporting information


**Data S1**. DISCOVER (Diagnostic Interview for Seizure Classification Outside of Video EEG Recording) questionnaires were administered to patients and external observers (if available) for each patient‐reported seizure type.

**Data S2**. Formulas used in analysis *k*, *p_pos_*,and *P_neg_*.
