# Supplementary material for: Investigation of patient and observer agreement on description of seizures at initial clinical visit
Source: Ann Clin Transl Neurol. 2019 Dec 5;6(12):2601–6. doi: 10.1002/acn3.50950 (PMC6917334; doi:10.1002/acn3.50950)
Supplement: Supplementary file 1 — Data S1. DISCOVER (Diagnostic Interview for Seizure Classification Outside of Video EEG Recording) questionnaires were administered to patients and external observers (if available) for each patient‐reported seizure type. [file ACN3-6-2601-s001.docx]

**DISCOVER form subsections included in Analysis**

**PART I: Questions for the patient**

SZ name (if given): ____________

# of seizures of this type in your lifetime? **☐** 1 **☐** 2-5 **☐** 6-9 **☐** 10-29 **☐** 30-49 **☐** 50-99 **☐** 100+

When this seizure is at its strongest, can you:

remember what happens during this seizure?

| **☐** Always | **☐** Sometimes | **☐** Never | **☐** Not Sure/Unknown |
| --- | --- | --- | --- |

Do people observe you doing any of the following during this seizure?

- - Altered awareness Notes: ______________________________
  - Decreased ability to respond Notes: ______________________________
  - Mouth movement (e.g. lip smacking) Notes: ______________________________
  - Drooling Notes: ______________________________
  - Glassy stare Notes: ______________________________
  - Eyes closed throughout Notes: ______________________________
  - Purposeless/aimless hand movements Notes: ______________________________
  - One hand/arm stiff or in an abnormal posture (**☐** Right ☐ Left ☐ Either ☐ Not Sure)
  - One side of the body stiff (**☐** Right ☐ Left ☐ Either ☐ Not Sure)
  - Both sides of the body stiff Notes: ______________________________
  - Falling to the ground/losing posture Notes: ______________________________
  - Talking nonsense Notes: ______________________________
  - Speaking repetitive phrases Notes: ______________________________
  - Other noises __________________
  - None of the above
  - Unknown/not sure

With this seizure, do people tell you that you stiffen and shake all over?

| **☐** Yes | **☐** Sometimes | **☐** Never | **☐** Not Sure/Unknown |
| --- | --- | --- | --- |

*If “Sometimes” is checked, make sure seizures with and without stiffening and shaking are recorded on two separate forms.*

*If “Never” or “Not sure/Unknown” is checked, please skip*

If so, how long does the shaking last? _______________________________

And report any of the following:

- - - Back arching Notes: ______________________________
    - Eyes closed Notes: ______________________________
    - Eyes rolled up Notes: ______________________________
    - Turns blue Notes: ______________________________
    - Drooling Notes: ______________________________
    - Bloody drool Notes: ______________________________
    - Prolonged groan or scream Notes: ______________________________
    - Grunting Notes: ______________________________
    - Other noise ______________________
    - None of the above
    - Unknown/not sure

When this seizure has ended, do you often find yourself in a different position or location than when this seizure started? Y N

If so, please describe:
__________________________________________________________________________________________________________________________________________

How do you feel after this seizure type? For how long?

- - Tired Duration:______________________
  - Confused Duration:______________________
  - Weak on one side (**☐** R **☐** L **☐** Either) Duration:______________________
  - Unable to speak clearly Duration:______________________
  - Sore in your muscles Duration:______________________
  - Breathing abnormally (deep, Duration:______________________

shallow, irregularly)

- - Other 1: __________________ Duration:______________________
  - Other 2: __________________ Duration:______________________
  - Immediately returns to normal
  - Unknown/not sure

**PART II: Questions for the observer**

SZ name (if given): _____________________________

How many of the patient’s seizures of this type have you witnessed? ______________________

**☐** 0 **☐** 1 **☐** 2-5 **☐** 6-9 **☐** 10+

Do you observe any of the following during this seizure?

- - Mouth movement (e.g. lip smacking) Notes: ______________________________
  - Drooling Notes: ______________________________
  - Glassy stare Notes: ______________________________
  - Eyes closed throughout Notes: ______________________________
  - Purposeless/aimless hand movements Notes: ______________________________
  - One hand/arm stiff or in an abnormal posture (**☐** Right ☐ Left ☐ Either ☐ Not Sure)
  - One side of the body stiff (**☐** Right ☐ Left ☐ Either ☐ Not Sure)
  - Both sides of the body stiff Notes: ______________________________
  - Falling to the ground/losing posture Notes: ______________________________
  - Talking nonsense Notes: ______________________________
  - Speaking repetitive phrases Notes: ______________________________
  - Other noises __________________
  - None of the above
  - Unknown/not sure

With this seizure type do you observe the patient stiffen and shake all over? Y N

If so, how long does the shaking last? _______________________________

And report any of the following:

**☐** Back arching Notes: ______________________________

- - - Eyes closed Notes: ______________________________
    - Eyes rolled up Notes: ______________________________
    - Turns blue Notes: ______________________________
    - Drooling Notes: ______________________________
    - Bloody drool Notes: ______________________________
    - Prolonged groan or scream Notes: ______________________________
    - Grunting Notes: ______________________________
    - Other noise ________________
    - None of the above
    - Unknown/not sure

Can the patient respond to you while they are having the seizure?

| **☐** Always | **☐** Sometimes | **☐** Never | **☐** Not Sure/Unknown |
| --- | --- | --- | --- |

Can the patient remember what happens during the seizure?

| **☐** Always | **☐** Sometimes | **☐** Never | **☐** Not Sure/Unknown |
| --- | --- | --- | --- |

When this seizure has ended, do you observe that the patient is often in a different position or location than when this seizure started? Y N

If so, please describe:
__________________________________________________________________________________________________________________________________________

After the seizure, do you observe any of the following?

**☐** Wakes up immediately Notes: ________________________

**☐** Looks as if in coma or deep sleep Notes: ________________________

**☐** Snoring or deep/irregular/loud Notes: ________________________

breathing

**☐** Decreased ability to respond Notes: ________________________
