# Supplementary material for: Investigation of patient and observer agreement on description of seizures at initial clinical visit
Source: Ann Clin Transl Neurol. 2019 Dec 5;6(12):2601–6. doi: 10.1002/acn3.50950 (PMC6917334; doi:10.1002/acn3.50950)
Supplement: Supplementary file 2 — Data S2. Formulas used in analysis k, ppos,and Pneg. [file ACN3-6-2601-s002.docx]

**Formulas Used in Analysis**

Because definitions may vary in different published reports, we provide below the formulas for Cohen’s Kappa (κ)^1^ as well as the proportion of positive agreement (p_pos_) and proportion of negative agreement (p_neg_)^2, 3^ used in this study.

|  | **Patient Responses** | |  |
| --- | --- | --- | --- |
| **Observer Responses** | **Yes** | **No** | **Totals** |
| **Yes** | A | B | G1 |
| **No** | C | D | G2 |
| **Totals** | F1 | F2 | N |

$$k=\frac{p_{o}-p_{e}}{1-p_{e}} Cohen^{'}s kappa value$$

$$p_{o}=\frac{A+D}{N} Observed proportion of agreement$$

$$p_{e}=\left( \frac{F1}{N} \right)\left( \frac{G1}{N} \right)+\left( \frac{F2}{N} \right)\left( \frac{G2}{N} \right) Proportion of agreement expected by chance$$

$$p_{pos}= \frac{A}{(F1+G1)/2} Proportion of positive agreement$$

$$P_{neg}=\frac{D}{(F2 +G2)/2} Proportion of negative agreement$$

**References**

1. Cohen J. A coefficient of agreement for nominal scales. Educational and Psychological Measurement 1960;20:37-46.

2. Feinstein AR, Cicchetti DV. High agreement but low kappa: I. the problems of two paradoxes. J Clin Epidemiol 1990;43: 543-548

3. Cicchetti DV, Feinstein AR. High agreement but low kappa: II. resolving the paradoxes. J Clin Epidemiol 1990;43:551-558.
